# The effects of salt consumption habits on iodine status and thyroid functions during pregnancy

**DOI:** 10.3906/sag-2007-127

**Published:** 2021-04-30

**Authors:** Mahmut APAYDIN, Taner DEMİRCİ, Özden ÖZDEMİR BAŞER, Bekir UÇAN, Mustafa ÖZBEK, Erman ÇAKAL

**Affiliations:** 1 Department of Endocrinology and Metabolism, Afyonkarahisar Health Sciences University, Afyonkarahisar Turkey; 2 Department of Endocrinology and Metabolism, Ministry of Health Yozgat City Hospital, Yozgat Turkey; 3 Department of Endocrinology and Metabolism, Faculty of Medicine, Sakarya University, Sakarya Turkey; 4 Department of Endocrinology and Metabolism, University of Health Sciences, Dışkapı Training and Research Hospital, Ankara Turkey

**Keywords:** Iodine, table salt, rock salt, pregnancy, TSH

## Abstract

**Background/aim:**

Iodine is the basic substrate for thyroid hormone synthesis and is vital for the general population and especially pregnant women. Iodine deficiency may cause severe health problems for a foetus. This study aimed to determine the relationship, if any, between iodine level and thyroid function tests, and to determine the relationship between consumption of salt types and its effects on thyroid function tests in the first trimester of pregnancy.

**Materials and methods:**

Three hundred and six pregnant women in the first trimester of pregnancy, who had known no thyroid disease history and had not received iodine supportive therapy, were included in the study. All patients were questioned for their preferred table salt or rock salt in daily use and urine iodine concentrations (UICs) were analysed in spot urine. The results were evaluated statistically according to salt usage preferences.

**Results:**

The median age of patients in the study was 27.8 (± 5.4). In terms of salt consumption habits, 235 (76.8%) of patients reported using table salt, and 71 (23.2%) reported using rock salt. Iodine deficiency was found in 75.81% (n = 232) of all cases according to urinalysis. Median UICs of table salt group were significantly higher than rock salt group (123.7 μg/L and 70.9 μg/L respectively, P < 0.001).

**Conclusion:**

Although large-scale salt iodination began long time ago, iodine deficiency is still a serious health problem among pregnant women. According to this study, use of rock salt is associated with low urinary iodine concentration in pregnant women and TSH values within the reference limits are not a good indicator for determining the iodine level.

## 1. Introduction 

Iodine is an essential element for thyroid hormone synthesis. Iodine requirements can be met by consuming iodine-containing nutrients or products with added iodine, such as table salt. When iodine intake is insufficient, thyroid hormone synthesis decreases and some related thyroid diseases may occur. For example, iodine deficiency in adults and adolescents can lead to certain thyroid diseases such as hypothyroidism, simple diffuse goitre, and nodular goitre [1].

Iodine deficiency during pregnancy can cause maternal and foetal hypothyroidism, goitre, congenital anomalies, mental retardation and cretinism [1]. Therefore, it is vital to maintain adequate iodine levels during pregnancy and the lactation period. Iodine deficiency is defined as a health problem, especially in developing countries, and its negative effects on child development can be prevented completely after replacement [2,3]. 

The tests which is being used to evaluate the level of iodine are urine iodine, serum thyroglobulin, thyroid gland sizes and the TSH level of a newborn [4–6]. The most commonly used and the most useful test is the urine iodine concentration. It can be used with urine samples taken at any time of day. Almost 90% of iodine taken into the body is detected in the urine. The World Health Organization (WHO) defined an adequate iodine level in urine at 100–300 μg/L for nonpregnant adults and children [7]. 

Iodine requirement increases in the early periods of pregnancy. There are many reasons for the increased need for iodine during pregnancy. The most important reason is to meet the thyroid hormone requirement of the foetus and to maintain maternal euthyroidism. Other reasons are to increase renal iodine clearance and placental deiodination [8]. For all of these reasons, adequate urine iodine level is defined as 150–250 μg/L during the pregnancy and lactation period. Iodine deficiency is defined as follows according to the UIC: 100–150 μg/L, 50–99 μg/L and <50 μg/L indicates mild, moderate and severe iodine deficiency in pregnancy, respectively [6,7,9].

A salt iodination program was started in the United States in 1922, and was implemented in Europe in the 1950s. In Turkey, the salt iodination program started in 1998. After legal arrangements, the Turkish food codex edible salt notification was issued, and the enrichment of table salts with iodine was made compulsory (25–40 mg/kg KIO3 as of 2010) [10].

The primary aim of this study is to determine iodine level and its relationship with thyroid function tests in first trimester of pregnancy. The secondary aim was to determine the effect of basic salt varieties on iodine status and thyroid function tests.

## 2. Material and methods

### 2.1. Subjects

This study was designed retrospectively and was conducted between December 2019 and March 2020. Three hundred and six healthy pregnant women in the first trimester were included in this study. The study was carried out in Yozgat city which is taking place in centre part of Turkey (The Central Anatolia Region) and is known as one of the iodine deficient areas in the country. Participants were selected in their outpatient applications for routine gynaecological evaluation. Written and signed voluntary consent forms were obtained in all cases. All patients were questioned about the basic salt types (table salt; iodized salt or rock salt; noniodized salt) they consumed daily. Serum TSH level was used for the evaluation of thyroid function tests, and cases within the reference range were accepted as having normal thyroid function. FT4 level was used to evaluate the differential diagnosis of possible secondary hypothyroidism. Cases with normal thyroid function tests in the first antenatal visit (4–12 weeks of pregnancy) were included in the study and the retrospective data were checked in the hospital information system. Those positive for thyroid autoantibody were not included in the study. In addition, pregnant women who have history of usage iodine-containing multivitamin or amiodarone tablets, having received any contrast radiographic examination within past 6 months and those undergoing salt-restricted diet therapy due to the presence of hypertension or other medical reasons were excluded.

In general, the hypothyroidism limit for pregnant women is accepted as TSH ≈ 4 mU/L according to the 2017 ATA criteria [11]. Treatment algorithm is usually designed according to this value. We analysed pregnant women with TSH values in the reference range presented for the adult population in 2 different groups as TSH <4 mU/L and ≥4 mU/L according to this threshold value.

This study has been approved by Yozgat Bozok University Ethics Committee and was performed in accordance with the ethical standards laid down in the 1964 Declaration of Helsinki and its later amendments. 

### 2.2. Laboratory evaluation

In our laboratory, TSH and free T4 (FT4) examinations were performed with the Unicell Dxl800 Hormone Analyzer device owned by Beckman Coulter by chemiluminescence method. The normal reference ranges of TSH and FT4 were 0.39–5.32 mU/L and 0.47–1.16 mU/L, respectively. Spot urine test was preferred for the determination of urine iodine level. The first urine of the day was evaluated for analysis. UIC in spot urine was measured with an Iodine Viem SR-I-100 device using the spectrophotometric method.

### 2.3. Statistical analysis

Statistical analyses were performed using SPSS version 22 software (IBM Corp., Armonk, NY, USA). The suitability of the variables to normal distribution was examined using visual (histogram and probability graphs) and analytical methods (Kolmogorov–Smirnov). According to the results of these analyses, the variables of TSH, FT4 and urinary iodine levels were not normally distributed, while the age variable was normally distributed. Descriptive analyses were performed using the median and interquartile range (using frequency tables for ordinal variables) for nonnormally distributed variables. The Mann–Whitney U test was used to compare the variables that were not normally distributed. Pearson’s chi-squared test was used to determine whether there was no difference between the groups in terms of quality variables. P values below 0.05 were considered statistically significant.

## 3. Results

**Figure 1:**
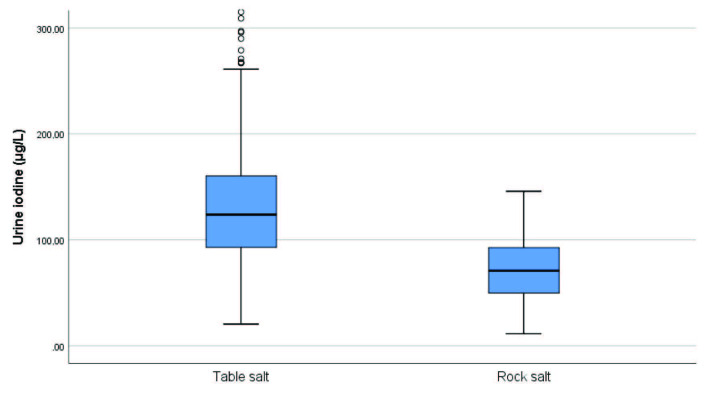
Comparison of urinary iodine level among those using table salt and rock salt.

**Figure 2:**
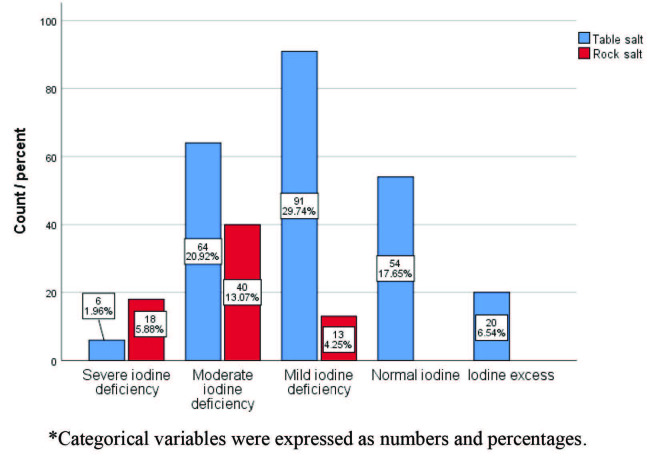
The distribution of salt consumption varieties according to iodine deficiency levels. *Categorical variables were expressed as numbers and percentages.

The mean age of the 306 patients in the study was 27.8 ± 5.4 years. In terms of salt consumption habits, 235 (76.8%) of the patients reported using table salt, while 71 (23.2%) reported using rock salt (Table 1). The median value of UIC was 112 μg/L. The median values ​​of urine iodine level in those using table salt and rock salt were 123.7 and 70.9, respectively (Table 2). When patients were grouped according to their salt consumption habits (table salt and rock salt) no differences were detected in serum TSH and FT4 levels (P = 0.301 and P = 0.793, respectively); however, there was a significant difference in urine iodine levels (P < 0.001) (Figure 1). Clinical classification was made according to urine iodine levels. According to this classification, only 17.65% of study patients (n = 54) had normal iodine levels and reported using table salt (Figure 2).

Participants grouped according to TSH level (TSH ≥4 mU/L and TSH <4 mU/L) were compared according to their salt consumption habits, there was no significant difference between the two groups (P = 0.69) (Table 3). However, the difference between urinary iodine levels in these two groups was found to be higher in the group with TSH≥ 4 (117.5 vs. 107.0 μg/L) and was statistically significant (P = 0.030) (Table 3).

**Table 1:** Descriptive statistics of basal characteristic features.

		Results*
Age (years)		27.85(±5.40)
UIC (μg/L)		112(11.63–714.00)
	In participants using table salt (n: 235)	123.70(20.69–714.00)
	In participants using rock salt (n: 71)	70.9(11.63–146.00)
TSH (mU/L)		2.75(0.39–5.32)
FT4 (mU/L)		0.74(0.47–1.16)

**Table 2:** Comparison of median UIC and thyroid function test results among those using table salt and rock salt.

	Results*		
	Table salt(n: 235)	Rock salt(n: 71)	P value
UIC (μg/L)	123.70(92.60–161.00)	70.90(49.68–93.00)	<0.001
TSH (mU/L)	2.50(1.40–3.70)	3.02(1.68–3.50)	0.301
FT4 (mU/L)	0.75(0.66–0.81)	0.73(0.63–0.85)	0.793

**Table 3:** Comparison of salt consumption varieties and urine iodine results by TSH level (4 mU/L).

	Results*		
	TSH < 4	TSH ≥ 4	P value
Table salt, n (%)	187 (76.3)	48 (78.7)	0.69
Rock salt, n (%)	48 (23.7)	23 (21.3)	
UIC (μg/L)	107.0	117.5	0.03

## 4. Discussion

Iodine deficiency is a common health problem in society. It is known that iodine deficiency in women during pregnancy and lactation periods causes foetal-neonatal neuronal development anomalies, foetal hypothyroidism and even causes fetal death in severe cases [3,12–15]. The median UIC we found (112 μg/L) is described as a mild iodine deficiency. It was slightly higher than that obtained in the other studies performed. For instance, two different studies which were evaluated the iodine requirement in pregnancy period conducted by François Delange et al. and Manousou et al. were shown that median UIC in the pregnancy groups were found to be 91 μg/L and 85 μg/L, respectively [6,16]. Another study conducted by Koyuncu et al. evaluated iodine level in 1st trimester pregnant women, the median UIC was 81.67 μg/L [17]. These results were far from the adequate iodine levels.

In a multicentre study, performed before the onset of salt iodination program in our country, Erdoğan et al., evaluated the prevalence of endemic goitre and iodine level in school-age children. As a result of study, adequate urinary iodine level (> 100 μg /L) was not detected any of the participants in this study centers [18]. In the 10th year of the salt iodination program, in the study evaluating urinary iodine level in school-age children conducted by Erdoğan et al., was stated that significant progress was made in removing iodine deficiency but the average iodine level was still insufficient especially in rural areas [19,20].

Despite the fact that a salt iodination program has been applied in Turkey for about 20 years, the use of noniodized salt still continues in approximately one of four pregnant women (23.2% of study patients) in our study. This rate was 4.7% in the study conducted by Mousa et al. (the rate of the group those who has not knowledge about used salt type in this study was 23.6%, and the group who used table salt was 71.7%), by Anaforoğlu et al., 10.5% of participants reported using rock salt [10,21]. Traditional and socioeconomic reasons were thought to cause this situation. These inquiries are very important because adequate iodine levels were not found in any of pregnant women who reported using rock salt, in our study. 

In our study, iodine deficiency was found in 68.5% (n = 161) of those who used table salt, and it was thought that the wrong habits in cooking and salt storage conditions could be caused. One of the possible limitations in our study was that these factors were not asked to the patients. In the study conducted by Anaforoğlu et al., it was found that 25% of the pregnant women kept salt in closed and invisible containers and 65% added salt after cooking, as appropriate [10]. 

According to the NHANES-III survey conducted in the United States between 1988 and 1994, despite the iodination policies implemented since the 1920s, the iodine concentration of 6.9% of pregnant women was still determined as 50 mcg/L. However, according to the same study, no relationship was found between serum TSH and FT4 levels and iodine concentrations of individuals [22]. In our study, when patients were grouped according to their TSH levels, it was found that salt consumption habits were not different from each other. Therefore, it can be interpreted that serum TSH level cannot predict iodine concentration well in pregnant women.

It is very important for healthy foetus development to start the iodine replacement even before conception, and to maintain adequate iodine level after pregnancy. Although there is a consensus on iodine replacement among pregnant women with severe and moderate iodine deficiency, there are various opinions about routine iodine replacement in those with mild deficiency and those living in iodine sufficient regions. The American Thyroid Association and the Endocrine Society recommend taking 150 μg/day of iodine supplementation during the pregnancy and lactation period [23,24]. In a study by Abel et al., iodine replacement was not recommended for pregnant women with suboptimal iodine intake (UIC 100–149 μg/day) [25]. The reason for this recommendation is may be that excessive iodine intake may lead to paradoxical hypothyroidism and this may lead to hyperthyroidism in pregnant women with multinodular goitre disease [23,26].

There may be some possible limitations in this study. Firstly, It was designed as a single-center study and UIC was evaluated in cross-sectionally. Other limitations such as, antenatal or postnatal fetal thyroid evaluation and maternal thyroid ultrasonographic imaging were not performed.

In conclusion **,** despite all salt iodination policies and related regulations, our current study showed that rock salt is still used widely in our country. Therefore, we think that these policies should be implemented more decisively. Also, according to the results of our study, TSH values within the reference range are not a good indicator of adequate iodine intake. Therefore, UIC or other iodine value determination tests may be done for providing adequate iodine level in early pregnancy period.

## Informed consent

This study was approved by the Local Ethics Committee of Yozgat Bozok University (Approval no. 2017-KAEK-189_2019.12.25_07). Informed consent forms were signed by all patients. Data were obtained for scientific purposes. 
